# Tau reduction attenuates autism-like features in *Fmr1* knockout mice

**DOI:** 10.1186/s13229-023-00574-1

**Published:** 2023-11-07

**Authors:** Shanshan Zhao, Xiangyu Jiang, Linkun Han, Yiru Jiang, Yong Wang, Jian Meng, Xiang Zhu, Xian Zhang, Hong Luo, Yun-wu Zhang

**Affiliations:** Xiamen Key Laboratory of Brain Center, The First Affiliated Hospital of Xiamen University, and Fujian Provincial Key Laboratory of Neurodegenerative Disease and Aging Research, Institute of Neuroscience, School of Medicine, Xiamen University, Xiamen, 361102 Fujian China

**Keywords:** Antisense oligonucleotide, Autism spectrum disorder, *FMR1*, Fragile X syndrome, P38/MAPK signaling, Tau

## Abstract

**Background:**

Fragile X syndrome (FXS) is a leading cause of autism spectrum disorder (ASD) and resulted from a loss of the *FMR1*-encoded fragile X messenger ribonucleoprotein 1 (FMRP) protein due to large CGG repeat expansions in the promoter region of the *FMR1* gene. The microtubule-associated protein Tau is a promising target for Tauopathic diseases and our preliminary study found that Tau protein levels were increased in the brain of *Fmr1* knockout (KO) mice, a model of FXS. However, whether Tau reduction can prevent autism-like features in *Fmr1* KO mice and become a novel strategy for FXS treatment remain unknown.

**Methods:**

Tau was genetically reduced in *Fmr1* KO mice through crossing *Fmr1*^±^ female mice with *Mapt*^±^ male mice. The male offspring with different genotypes were subjected to various autism-related behavioral tests, RNA sequencing, and biochemical analysis. *Fmr1* KO male mice were treated with Tau-targeting antisense oligonucleotide (ASO) and then subjected to behavioral tests and biochemical analysis.

**Results:**

Tau expression was increased in the cortex of *Fmr1* KO mice. Genetically reducing Tau prevented social defects, stereotyped and repetitive behavior, and spine abnormality in *Fmr1* KO mice. Tau reduction also reversed increased periodic activity and partially rescued *Per1* expression reduction in *Fmr1* KO mice. Moreover, Tau reduction reversed compromised P38/MAPK signaling in *Fmr1* KO mice. Finally, Tau-targeting ASO also effectively alleviated autism-like phenotypes and promoted P38/MAPK signaling in *Fmr1* KO mice.

**Limitations:**

Our study is limited to male mice, in agreement with the higher incidence of FXS in males than females. Whether Tau reduction also exerts protection in females deserves further scrutiny. Moreover, although Tau reduction rescues impaired P38/MAPK signaling in *Fmr1* KO mice, whether this is the responsible molecular mechanism requires further determination.

**Conclusion:**

Our data indicate that Tau reduction prevents autism-like phenotypes in *Fmr1* KO mice. Tau may become a new target for FXS treatment.

**Supplementary Information:**

The online version contains supplementary material available at 10.1186/s13229-023-00574-1.

## Background

Fragile X syndrome (FXS) is a one of the most common autism spectrum disorders (ASDs) and results from mutations in the X-linked *FMR1* gene: the 5’ untranslated region of *FMR1* contains a CGG-triplet repeat, whose abnormal expansion (> 200 repeats) can cause epigenetic silencing of *FMR1* and thus a loss of *FMR1*-encoded fragile X messenger ribonucleoprotein 1 (FMRP). FXS patients exhibit a series of cognitive and behavioral deficits, such as intellectual disability, language impairment, social defects, and stereotyped and repetitive behavior [1–3]. Currently there is no effective treatment for FXS and it is urgent to identify new targets for therapeutic strategy development.

Tau is a microtubule-associated protein and encoded by the *MAPT* gene. Tau is highly enriched in neurons and regulates physiological functions of microtubule and synapses. Dysregulated modifications of Tau and/or mutations of *MAPT* underlie the pathogenesis of Tauopathy that includes Alzheimer’s disease, frontotemporal dementia, Pick’s disease, etc., making Tau a promising target for Tauopathy treatment [4–6]. Moreover, several recent studies have shown that genetic ablation of Tau and Tau reduction can attenuate autism-like phenotypes in *Cntnap2*^*−/−*^ mice and Dravet syndrome model mice (*Scn1a*^*RX/*+^) [7–10]. Nevertheless, whether Tau reduction exerts protection for FXS, a leading cause of ASD, remains unknown.

## Materials and methods

### Animals

*Fmr1* (FVB background) and *Mapt* (C57BL/6/SV129 background) knockout (KO) mice were kind gifts from Drs. Chen Zhang and Peng Lei, respectively [11, 12]. Mice were maintained at 20–25 °C with a 12 h light/dark cycle and with free access to food and water at Laboratory Animal Center of Xiamen University. *Fmr1*^±^ female mice were mated with *Mapt*^±^ male mice to obtain wild type (WT), *Mapt*^±^, *Fmr1*^*−/y*^, and *Fmr1*^*−/y*^;*Mapt*^±^ male offspring (in FVB;C57BL/6/SV129 mixed background) that were used for following studies.

### Quantitative real-time PCR (qRT-PCR)

Total mRNAs were isolated from tissues of the hippocampus and the entire cerebral cortex using TRIzol reagent (ThermoFisher) and transcribed into cDNA using the Rever Tra Ace qPCR RT Kit (TOYOBO). qRT-PCR was performed using the FastStart Universal SYBR Green Master (ROX). Primer sequences for target genes were as follows: *Fmr1*, forward-5′-TTTCGAAGTCTGCGCACCAA-3′, reverse-5′-CACTGCATCTTGATCCTCTCCAT-3′; *Mapt*, forward-5′-ACTGAGAACCTGAAGCACCA-3′, reverse-5′-GGATGTTCCCTAACGAGCCA-3′; *Per1*, forward-5′-CAAACGGGATGTGTTTCGGG-3′, reverse-5′-GTTAGGCGGAATGGCTGGTA-3′; β-actin, forward-5′-AGCCATGTACG TAGCCATCCA-3′, reverse-5′-TCTCCGGAGTCC ATCACAATG-3′.

### Behavioral tests

By crossing *Fmr1*^±^ female mice (FVB background) with *Mapt*^±^ male mice (C57BL/6/SV129 background), we acquired the offspring with an FVB;C57BL/6/SV129 mixed background. Because FXS afflicts males much more than females and disease phenotypes are already obvious in pediatric and adolescent patients [1–3], we selected the male offspring at 1 month of age, which is roughly equivalent to 3.5-year-old and 12.5-year-old humans based on total life span and based on maturational rate comparisons, respectively (LIFE SPAN AS A BIOMARKER: https://www.jax.org/research-and-faculty/research-labs/the-harrison-lab/gerontology/life-span-as-a-biomarker) [13], for the following behavioral tests.

Open field test: mice were individually placed in the center of an open field (40 × 40 × 40 cm) and allowed to explore freely for 10 min. Total travel distance, time spent in the center, and center entry numbers were recorded for analysis [14–16].

Three-chamber social interaction test: this test was performed in a rectangular box with three chambers. There was an empty cage in each side chamber. The test mouse was first allowed to habituate in the three chambers for 10 min, and then moved into the middle chamber. After placing a strange mouse (stranger 1) into one of the empty cages, the test mouse was allowed to explore freely for 10 min. Then, another strange mouse (stranger 2) was placed into the other empty cage and the test mouse was allowed to explore freely for another 10 min. Mouse movement and contact time with the cage were recorded for analysis [15, 16].

Self-grooming test: the test mouse was placed in a clean cage lined with fresh bedding. After 5 min of habituation, mouse was recorded for its spontaneous behavior for 10 min and the time spent grooming and bout numbers were analyzed [15, 16].

Nest building test: the test mouse was placed in a cage containing fresh bedding and a square piece of cotton (3 g). The next morning, the nest was scored in a 1–5 scale: score 1, 90% nesting cotton remains intact; score 2, 50–90% of nesting cotton remains intact; score 3, 50% nesting cotton was shredded and spread around the cage; score 4, 90% of nesting cotton was torn and clustered together; score 5, 90% of nesting cotton was torn and reformed into a (nearly) perfect nest [15–17].

Autonomous wheel-running test: the test mouse was placed in a cage equipped with a running wheel one day in advance for habituation, and then recorded for the activity for 5 consecutive days in constant darkness environment [18–20].

### Golgi staining

Golgi staining was performed following previously reported protocols [14, 15]. Briefly, mouse brains were dissected, sliced into coronal slices (150 μm thick), and stained using FD Rapid Golgi Stain Kit (FD Neuro Technologies). Images of the secondary apical dendrites of cortical layer II/III neurons were acquired with an Olympus FV1000MPE-B confocal microscope. Spine density and ratios of mature (mushroom shape) and immature (thin and stubby shape) spines were analyzed.

### RNA sequencing and analysis

Total mRNAs derived from mixed cortical and hippocampal tissues of studied mice were subjected to standard RNA sequencing and data cleaning by Beijing Genomics Institute (BGI). Differentially expressed genes (DEGs) were determined with an absolute value of log_2_(fold change) ≥ 0 and a *Q*-value ≤ 0.05. Analysis of Gene Ontology (GO) enrichment was performed on BGI’s Dr. Tom platform (https://report.bgi.com).

### Western blotting

Protein lysates of tissues of the hippocampus and the cerebral cortex of studied mice were subjected to SDS-PAGE, transferred to PVDF membranes, and then incubated sequentially with indicated primary antibodies, appropriated HRP-conjugated secondary antibodies, and enhanced chemiluminescence reagents for protein band development. Primary antibodies used were: anti-Tau (Tau5, Invitrogen, #AHB0042, 1:1000), anti-FMRP (CST, #4317S, 1:1000), ant-p-S6 (Ser240/244, CST, #5364S, 1:1000), anti-S6 (CST, #2217S, 1:1000), anti-p-AKT (Ser473, CST, #9271S, 1:1000), anti-AKT (CST, #9272S, 1:1000), anti-p-mTOR (Ser2448, CST, #5536S, 1:1000), anti-mTOR (CST, #2983S, 1:1000), anti-p-ERK1/2 (Thr202/Tyr204, CST, #4370S, 1:1000), anti-ERK1/2 (CST, #4695S, 1:1000), anti-p-P38 (Thr180/Tyr182, Proteintech, #28796-1-AP, 1:1000), anti-P38 (CST, #8690S, 1:1000), anti-β-actin (CST, #8457S, 1:10000), and anti-GAPDH (Abclonal, #AC001, 1:10000). Secondary antibodies used were: Goat anti-Rabbit IgG (H + L)-HRP (ThermoFisher, #31460, 1:4000) and Goat anti-Mouse IgG (H + L)-HRP (ThermoFisher, #31430, 1:4000).

### Mouse Tau antisense oligonucleotides (ASOs) and treatment

The sequences of mouse Tau ASO (5′-ATCACTGATTTTGAAGTCCC-3′) and scrambled ASO (5′-CCTTCCCTGAAGGTTCCTCC-3′) and their modifications followed a previous study [21]. For treatment, *Fmr1* KO male mice (FVB background) at four weeks old were anesthetized and 14-day osmotic intracerebroventricular (ICV) pumps (RWD) with ASO were implanted subcutaneously on mouse back. The catheter was placed in the right lateral ventricle using the coordinates based on bregma: − 0.5 mm posterior, − 1.0 mm lateral, − 2.5 mm ventral [21, 22]. Catheters were removed two weeks later. Mice were allowed for another two-week recovery and then subjected to above-mentioned behavioral tests.

### Statistics

Statistical analyses were performed with GraphPad Prism 8 software. Unpaired t test was used for comparison between two groups. One-way analysis of variance (ANOVA) with Tukey’s post hoc test or two-way ANOVA with Bonferroni's post hoc test were used for multiple group comparisons. *p* < 0.05 was considered to be statistically significant. Data are presented as the mean ± SEM.

## Results

### Tau expression is increased in the cortex of Fmr1 KO mice

**Fig. 1:**
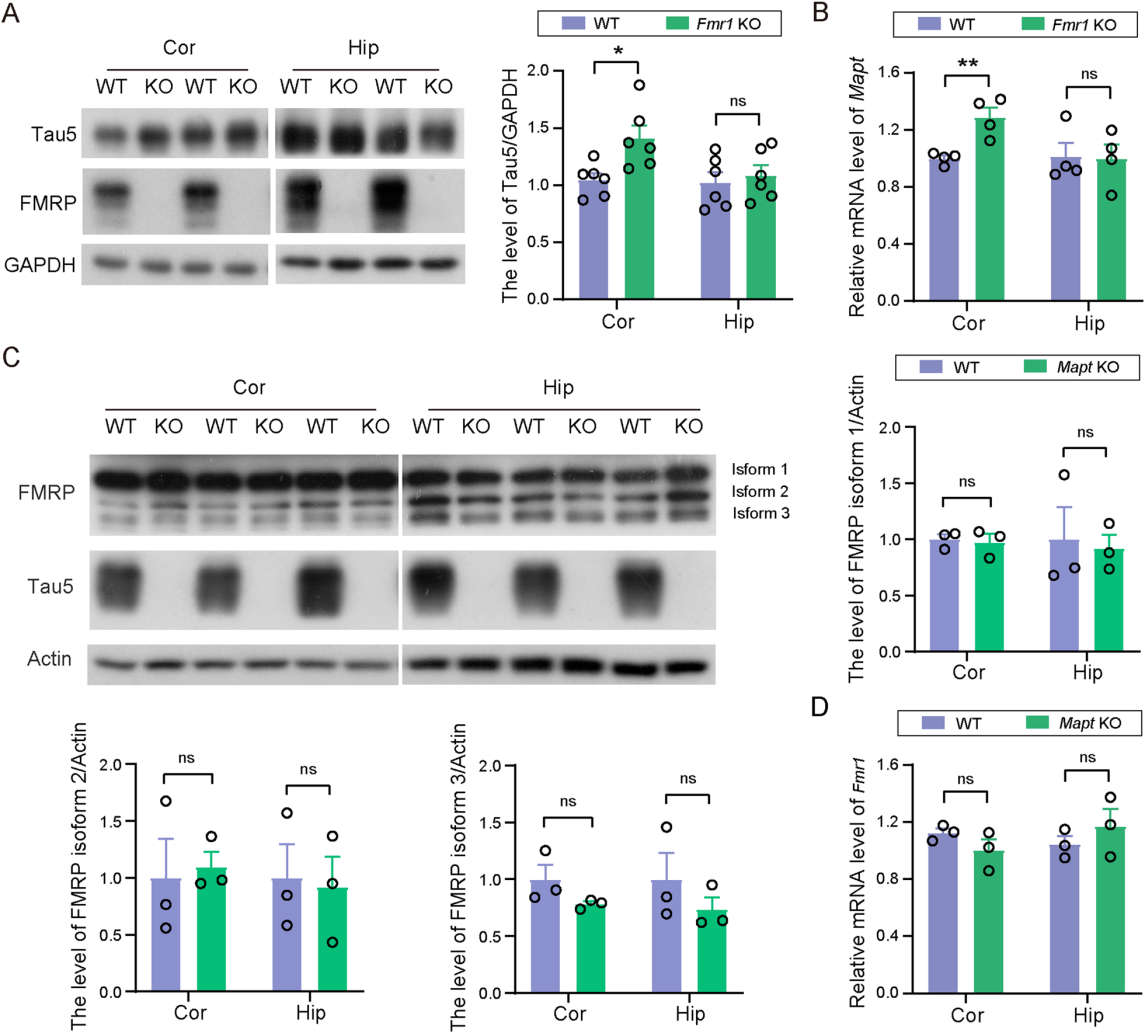
Tau and *Mapt* mRNA levels are increased in the cortex of *Fmr1* KO mice. **A** Tau protein in the cortex and hippocampus of WT and *Fmr1* KO mice (FVB background, 1.5-month-old) was detected by western blotting; and protein levels were quantified by densitometry for comparison after normalizing to those of GAPDH. WT: *n* = 6; *Fmr1* KO: *n* = 6. **B** *Mapt* mRNA levels in the cortex and hippocampus of WT and *Fmr1* KO mice (FVB background, 1.5-month-old) were detected by qRT-PCR and compared after normalizing to those of β-actin. WT: *n* = 4; *Fmr1* KO: *n* = 4. **C** FMRP protein was detected by western blotting in the cortex and hippocampus of WT and *Mapt* KO mice (C57BL/6/SV129 background, 4-month-old); and protein levels of three major FMRP isoforms (1, 2, and 3) were individually quantified by densitometry for comparison after normalizing to those of β-actin. WT: *n* = 3; *Mapt* KO: *n* = 3. **D** *Fmr1* mRNA levels in the cortex and hippocampus of WT and *Mapt* KO mice (C57BL/6/SV129 background, 4-month-old) were detected by qRT-PCR and compared after normalizing to those of β-actin. WT: *n* = 3; *Mapt* KO: *n* = 3. Unpaired t test. ns: not significant; *p < 0.05, **p < 0.01

We first detected and found that both Tau protein (Cor, *t*_(16)_ = 2.565, *p* = 0.0207, unpaired t test, Fig. 1A) and *Mapt* mRNA (Cor, *t*_(6)_ = 4.096, *p* = 0.0064, unpaired t test, Fig. 1B) levels were significantly increased in the cortical but not in the hippocampal regions of *Fmr1* KO mice when compared to WT controls. These results suggest that *Fmr1* regulates the expression of *Mapt* and thus tau protein levels. On the other hand, neither FMRP protein nor *Fmr1* mRNA levels were altered in the cortical and hippocampal regions of *Mapt* KO mice (Fig. 1C, D).

### Genetically reducing Tau attenuates autism-like behaviors in Fmr1 KO mice

**Fig. 2:**
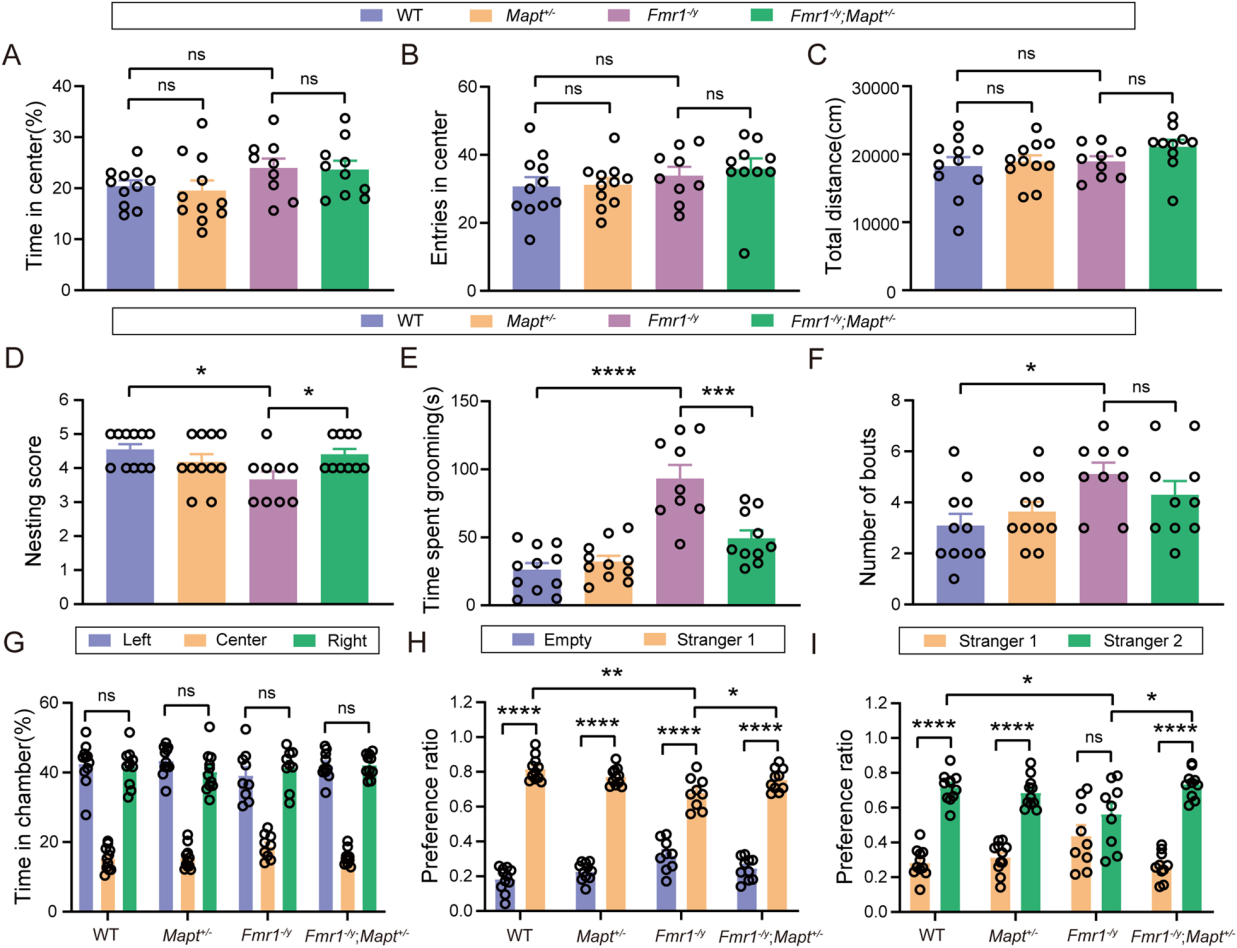
Genetically reducing Tau prevents autism-like behaviors in *Fmr1*^*−/y*^ mice. **A**–**I** *Fmr1* KO mice and *Mapt* KO mice were crossed and the offspring (1-month-old, FVB;C57BL/6/SV129 mixed background) were studied for their behaviors. In the open field test, time spent in the center (**A**), center entry numbers (**B**), and total travel distance (**C**) were analyzed. In the nest building test, nesting scores were analyzed (**D**). In the self-grooming test, grooming time (**E**) and bout numbers (**F**) were analyzed. In the three-chamber social interaction test, time spent in each chamber (**G**), time spent interacting with a Stranger 1 mouse and the empty cage (**H**), and time spent interacting with the Stranger 1 mouse and a Stranger 2 mouse (**I**) were analyzed. WT: *n* = 11; *Mapt*^±^: *n* = 11; *Fmr1*^*−/y*^: *n* = 9; *Fmr1*^*−/y*^;*Mapt*^±^: *n* = 10. One-way ANOVA followed by Tukey’s post hoc test for (**A**–**F**). Two-way ANOVA followed by Bonferroni’s post hoc test for G-I. ns: not significant, *p < 0.05, **p < 0.01, ***p < 0.001, ****p < 0.0001

We crossed *Fmr1*^±^ female mice with *Mapt*^±^ male mice to obtain WT, *Mapt*^±^, *Fmr1*^*−/y*^, and *Fmr1*^*−/y*^;*Mapt*^±^ male offspring. These mice were studied for their behaviors at 1 month of age. In the open field test, we found that time spent in the center, entries into the center and total travel distance were not different among the four groups of mice (Fig. 2A–C). In the nest building test, *Fmr1*^*−/y*^ mice had poorer nesting scores than those of WT mice, whereas *Fmr1*^*−/y*^;*Mapt*^±^mice had better nesting scores than *Fmr1*^*−/y*^ mice (*F*_(3,37)_ = 3.559, *p* = 0.0112 for *Fmr1*^*−/y*^ versus WT, *p* = 0.0470 for *Fmr1*^*−/y*^;*Mapt*^±^ versus *Fmr1*^*−/y*^, one-way ANOVA followed by Tukey’s post hoc test, Fig. 2D). In the self-grooming test, the grooming time (*F*_(3,37)_ = 21.46, *p* < 0.0001 for *Fmr1*^*−/y*^ versus WT, *p* = 0.0002 for *Fmr1*^*−/y*^;*Mapt*^±^ versus *Fmr1*^*−/y*^, one-way ANOVA followed by Tukey’s post hoc test, Fig. 2E) and bout numbers (*F*_(3,37)_ = 3.455, *p* = 0.0211 for *Fmr1*^*−/y*^ versus WT, one-way ANOVA followed by Tukey’s post hoc test, Fig. 2F) of *Fmr1*^*−/y*^ mice were significantly increased, whereas Tau reduction reversed the increased grooming time in *Fmr1*^*−/y*^ mice. In the three-chamber social interaction test, none of the four groups of mice showed preference for each chamber during the habituation phase (Fig. 2G). During the social preference testing phase, although all four groups of mice interacted more with a stranger mouse (Stranger 1) than an empty cage, *Fmr1*^*−/y*^ mice had less preference ratios to Stranger 1 than WT and *Fmr1*^*−/y*^;*Mapt*^±^mice (*F*_(3,74)_ = 13.29, *p* < 0.0001 for Empty versus Stranger 1 in all four groups, *p* = 0.0012 for Stranger 1 in *Fmr1*^*−/y*^ versus Stranger 1 in WT, *p* = 0.0497 for Stranger 1 in *Fmr1*^*−/y*^;*Mapt*^±^ versus Stranger 1 in *Fmr1*^*−/y*^, two-way ANOVA followed by Bonferroni’s post hoc test, Fig. 2H). During the social novelty testing phase, *Fmr1*^*−/y*^ mice showed no preference for a novel stranger mouse (Stranger 2) when compared to the familiar Stranger 1. While both WT and *Fmr1*^*−/y*^;*Mapt*^±^mice not only showed preference for Stranger 2 when compared to Stranger 1, but also had more preference ratios to Stranger 2 than *Fmr1*^*−/y*^ mice (*F*_(3,74)_ = 8.640, *p* < 0.0001 for Stranger 2 versus Stranger 1 in WT, *Mapt*^±^, and *Fmr1*^*−/y*^;*Mapt*^±^ groups, *p* = 0.0238 for Stranger 2 in *Fmr1*^*−/y*^ versus Stranger 2 in WT, *p* = 0.0161 for Stranger 2 in *Fmr1*^*−/y*^;*Mapt*^±^ versus Stranger 2 in *Fmr1*^*−/y*^, two-way ANOVA followed by Bonferroni’s post hoc test, Fig. 2I). Together, these results indicate that Tau reduction can ameliorate social defects and stereotyped and repetitive behavior in *Fmr1* KO mice.

### Tau reduction reverses spine abnormality in Fmr1 KO mice

**Fig. 3:**
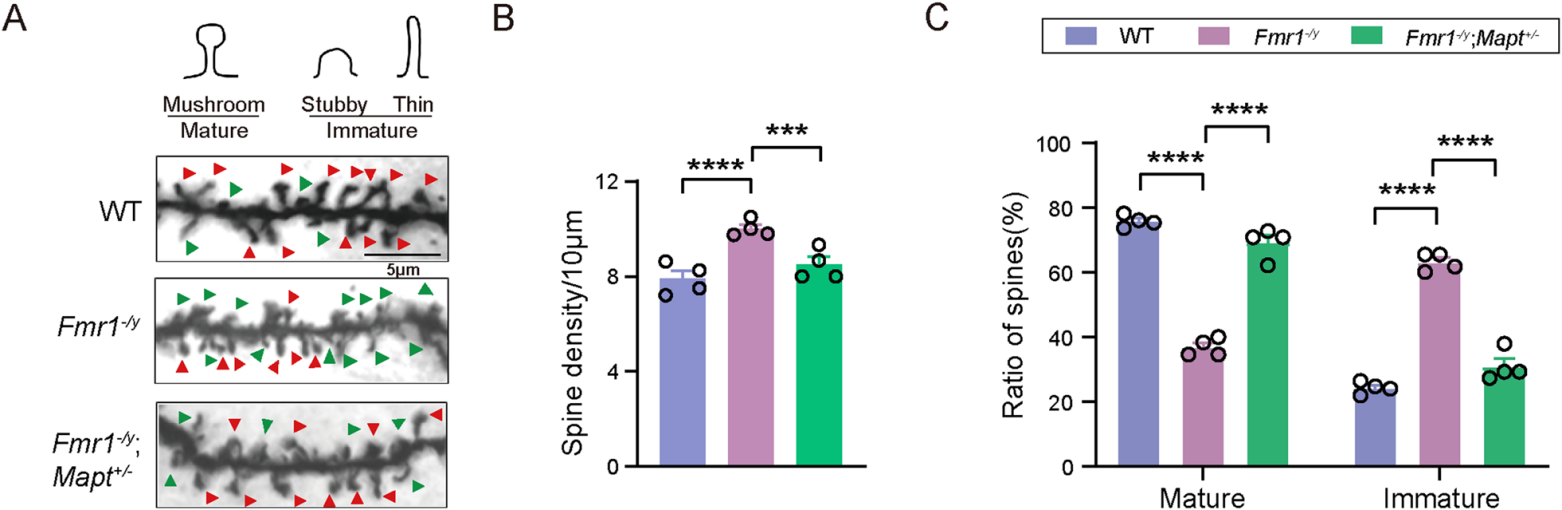
Genetically reducing Tau reverses spine abnormality in *Fmr1*^*−/y*^ mice. **A** Representative projection images of dendritic spines from cortical neurons from WT, *Fmr1*^*−/y*^, and *Fmr1*^*−/y*^;*Mapt*^±^ mice (2.5-month-old, FVB;C57BL/6/SV129 mixed background). Mature and immature dendritic spines were indicated by red and green arrowheads, respectively. Scale bars: 5 μm. **B**, **C** Comparisons of spine density (**B**) and ratios of mature and immature spines (**C**) of cortical neurons. One-way ANOVA followed by Tukey’s post hoc test. WT: *n* = 4; *Mapt*^±^: *n* = 4; *Fmr1*^*−/y*^: *n* = 4; *Fmr1*^*−/y*^;*Mapt*^±^: *n* = 4. ***p < 0.001, ****p < 0.0001

In the cortex of FXS patients and *Fmr1* KO mice, spine density is found to be increased and accompanied by decreased mature spines and increased immature spines [23–25]; and this may underlie dysregulated neuronal functions and abnormal behaviors in FXS [26]. We conducted Golgi staining and confirmed significantly increased spine density (*F*_(3,58)_ = 35.84, *p* < 0.0001 for *Fmr1*^*−/y*^ versus WT, *p* = 0.0006 for *Fmr1*^*−/y*^;*Mapt*^±^ versus *Fmr1*^*−/y*^, one-way ANOVA followed by Tukey’s post hoc test, Fig. 3A, B) and numbers of immature spines, as well as decreased mature spines (*F*_(2,9)_ = 155.3, *p* < 0.0001 for all comparisons, one-way ANOVA followed by Tukey’s post hoc test, Fig. 3A, C) in cortical neurons of *Fmr1*^*−/y*^ mice. Importantly, these alterations were reversed in *Fmr1*^*−/y*^;*Mapt*^±^ mice (Fig. 3A–C).

### Tau reduction reverses altered periodic activity in Fmr1 KO mice

**Fig. 4:**
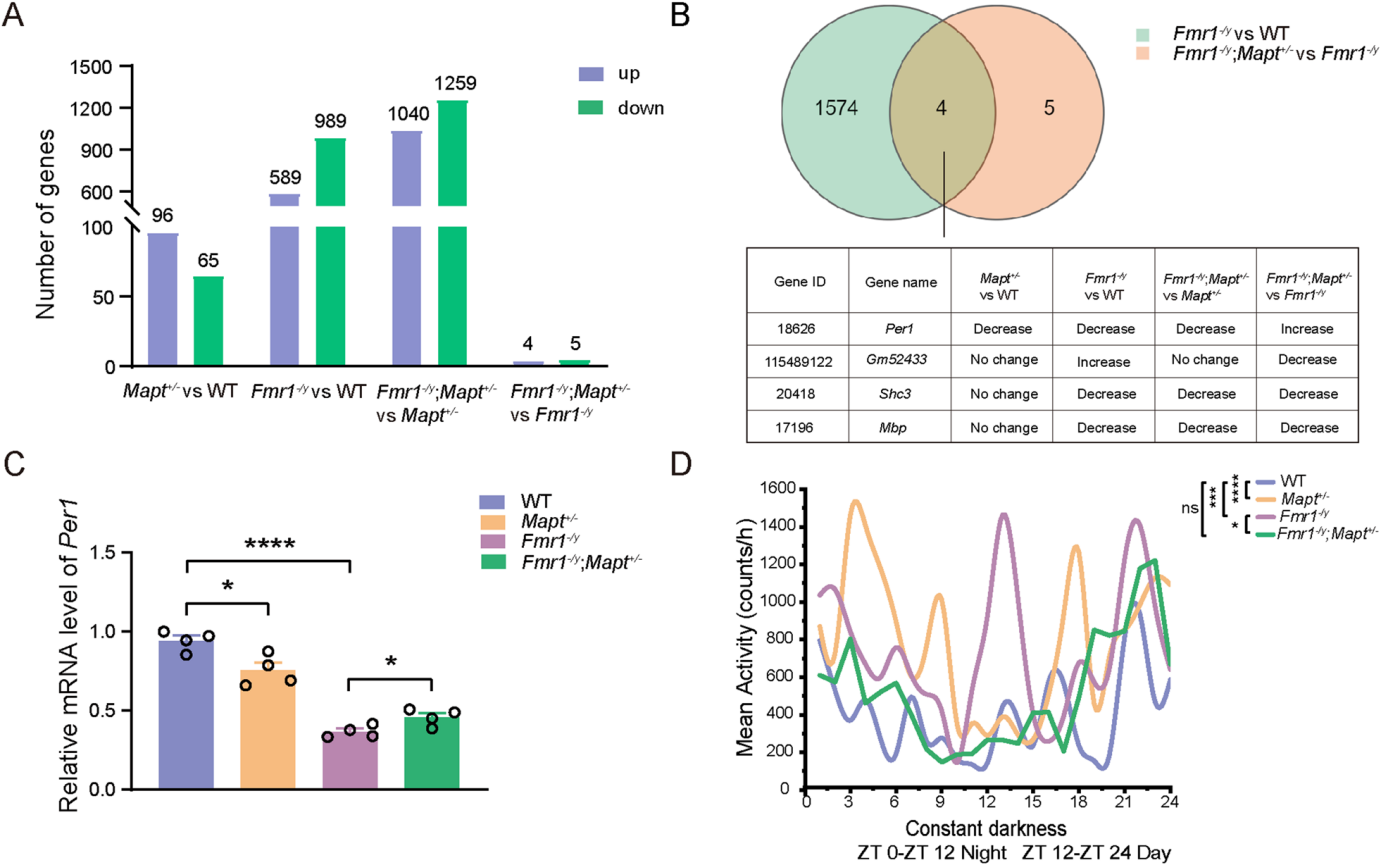
Genetically reducing Tau rescues circadian rhythm defect in *Fmr1*^*−/y*^ mice. **A** RNA sequencing identified DEGs between mice with different genotypes (FVB;C57BL/6/SV129 mixed background, 2.5 month-old). **B** DEGS shared by *Fmr1*^*−/y*^ versus WT and *Fmr1*^*−/y*^;*Mapt*^±^ versus *Fmr1*^*−/y*^ groups and their information. **C** *Per1* mRNA levels in the brain of mice with different genotypes were determined by qRT-PCR for comparison. One-way ANOVA followed by Tukey’s post hoc test. WT: *n* = 4; *Mapt*^±^: *n* = 4; *Fmr1*^*−/y*^: *n* = 4; *Fmr1*^*−/y*^;*Mapt*^±^: *n* = 4. **D** In the autonomous wheel-running test, the activities of different groups of mice were recorded for 5 consecutive days in a constant darkness environment and compared. Two-way repeated-measures ANOVA. WT: *n* = 3; *Mapt*^±^: *n* = 3; *Fmr1*^*−/y*^: *n* = 3; *Fmr1*^*−/y*^;*Mapt*^±^: *n* = 3. ns: not significant, *p < 0.05, ***p < 0.001, ****p < 0.0001

By performing RNA sequencing using a mixture of hippocampal and cortical tissues, we identified 96 upregulated and 65 downregulated DEGs in *Mapt*^±^ versus WT, 589 upregulated and 989 downregulated DEGs in *Fmr1*^*−/y*^ versus WT, 1040 upregulated and 1259 downregulated DEGs in *Fmr1*^*−/y*^;*Mapt*^±^ versus *Mapt*^±^, and 4 upregulated and 5 downregulated DEGs in *Fmr1*^*−/y*^;*Mapt*^±^ versus *Fmr1*^*−/y*^ (Fig. 4A).

With an assumption that genes responsible for Tau reduction-exerted protection would be those whose expressions are altered in *Fmr1*^*−/y*^ versus WT and reversed in *Fmr1*^*−/y*^;*Mapt*^±^ versus *Fmr1*^*−/y*^, we first determined DEGs that were shared by *Fmr1*^*−/y*^ versus WT and *Fmr1*^*−/y*^;*Mapt*^±^ versus *Fmr1*^*−/y*^. However, we only found 4 DEGs overlapped in the two groups as *Per1*, *Gm52433*, *Shc3*, and *Mbp* (Fig. 4B). Among the 4 DEGs, only the expressions of *Per1* and *Gm52433* showed opposite direction change between *Fmr1*^*−/y*^ versus WT and *Fmr1*^*−/y*^;*Mapt*^±^ versus *Fmr1*^*−/y*^, whereas the expression change directions were the same for *Shc3* and *Mbp* in the two groups.

*Per1* is an important circadian rhythm gene [27]. Since it is reported that FXS patients and animal models also exhibit abnormal circadian behavioral rhythm [28, 29], we further studied *Per1* mRNA expression by qRT-PCR. Consistent with RNA sequencing data, we found that *Per1* mRNA expression was decreased in *Fmr1*^*−/y*^ mice and partially reversed by *Mapt* deficiency (*F*_(3,12)_ = 63.12, *p* < 0.0001 for *Fmr1*^*−/y*^ versus WT, *p* = 0.0308 for *Fmr1*^*−/y*^;*Mapt*^±^ versus *Fmr1*^*−/y*^, one-way ANOVA followed by Tukey’s post hoc test, Fig. 4C). In the autonomous wheel-running test performed under constant darkness, we found that *Fmr1*^*−/y*^ mice had more periodic activity than WT mice, especially at the time around the night-day transition (*F*_(3,192)_ = 11.37, *p* = 0.0001 for *Fmr1*^*−/y*^ versus WT, two-way repeated-measures ANOVA, Fig. 4D); this is implies a circadian rhythm defect in *Fmr1*^*−/y*^ mice. While the periodic activity of *Fmr1*^*−/y*^;*Mapt*^±^ mice were less than *Fmr1*^*−/y*^ mice and comparable to WT mice (*F*_(3,192)_ = 11.37, *p* = 0.0270 for *Fmr1*^*−/y*^;*Mapt*^±^ versus *Fmr1*^*−/y*^, two-way repeated-measures ANOVA, Fig. 4D).

Interestingly, we noticed that *Mapt*^±^ mice also had decreased *Per1* mRNA expression (*F*_(3,12)_ = 63.12, *p* = 0.0176 for *Mapt*^±^ versus WT, one-way ANOVA followed by Tukey’s post hoc test, Fig. 4C) and increased periodic activity in the autonomous wheel-running test when compared to WT mice (*F*_(3,192)_ = 11.37, *p* < 0.0001 for *Mapt*^±^ versus WT, two-way repeated-measures ANOVA, Fig. 4D). Therefore, the rescuing effect of Tau reduction on autism-like phenotypes in *Fmr1* KO mice is unlikely through reversing *Per1* expression and periodic activity defect.

### Tau reduction reverses impaired P38/MAPK signaling in Fmr1^−/y^ mice

**Fig. 5:**
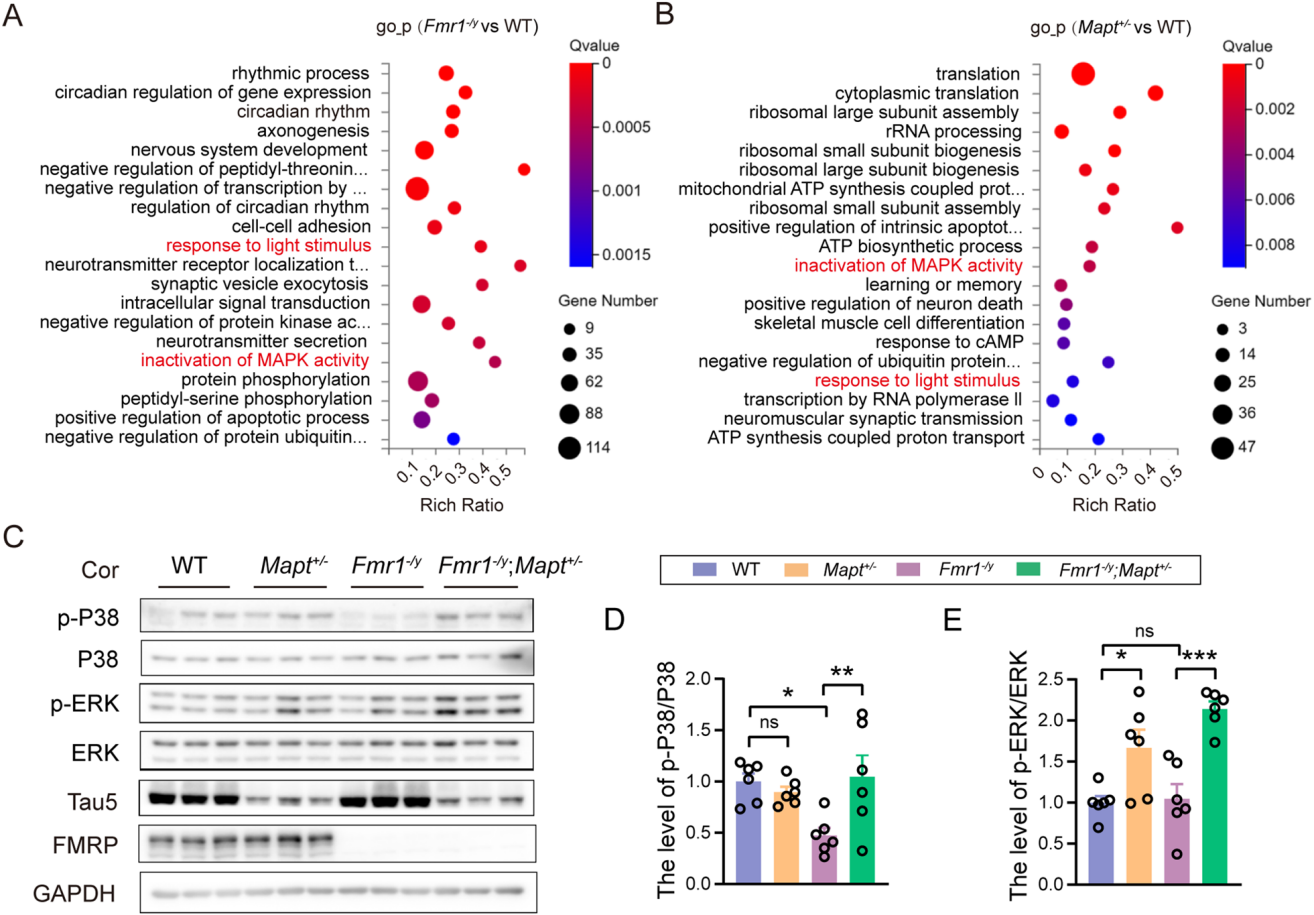
Genetically reducing Tau rescues impaired P38/MAPK signaling in *Fmr1*^*−/y*^ mice. **A**, **B** GO process enrichment analysis based on DEGs identified in *Fmr1*^*−/y*^ versus WT (**A**) and in *Mapt*^±^ versus WT (**B**) groups. Red ones indicate GO processes shared in (**A**) and (**B**). **C**–**E** Equal amounts of protein lysates from mouse cortical tissues were analyzed by western blotting for indicated proteins (**C**). Levels of phosphorylated p38 (**D**) and ERK (**E**) in different groups of mice (FVB;C57BL/6/SV129 mixed background, 2.5-month-old) were normalized to respective total protein levels for comparison. One-way ANOVA with Tukey’s post hoc test. WT: *n* = 6; *Mapt*^±^: *n* = 6; *Fmr1*^*−/y*^: *n* = 6; *Fmr1*^*−/y*^;*Mapt*^±^: *n* = 6. ns: not significant; *p < 0.05, **p < 0.01, ***p < 0.001

Since Tau deficiency minimally affected gene expression in *Fmr1* KO mice, we wondered whether gene processes/pathways affected by Tau deficiency could balance those affected by *Fmr1* deficiency and thereby providing the protection. By comparing the top 20 GO processes enriched with DEGs found in *Fmr1*^*−/y*^ versus WT and those found in *Mapt*^±^ versus WT (Fig. 5A, B), we found two overlapped GO processes: “response to light stimulus” and “inactivation of MAPK activity”, of which the former is related to circadian rhythm.

We then detected levels of MAPK pathway-related proteins in cortical tissues and found that levels of phosphorylated P38 were significantly decreased in *Fmr1*^*−/y*^ mice when compared to WT mice; and this decrease was reversed in *Fmr1*^*−/y*^;*Mapt*^±^ mice (*F*_(3,20)_ = 4.689, *p* = 0.0174 for *Fmr1*^*−/y*^ versus WT, *p* = 0.0094 for *Fmr1*^*−/y*^;*Mapt*^±^ versus *Fmr1*^*−/y*^, one-way ANOVA followed by Tukey’s post hoc test, Fig. 5C, D). While P38 phosphorylation was not different between *Mapt*^±^ and WT mice. The ERK signaling is another important MAPK pathway. However, although some studies reported increased ERK phosphorylation in *Fmr1* KO mice [30–32], other work suggested no change or even decrease of ERK phosphorylation [33–35]. Herein, we found that ERK phosphorylation was not altered in *Fmr1*^*−/y*^ mice when compared to WT mice but was significantly increased when Tau was genetically reduced (*F*_(3,20)_ = 12.26, *p* = 0.0194 for *Mapt*^±^ versus WT, *p* = 0.0002 for *Fmr1*^*−/y*^;*Mapt*^±^ versus *Fmr1*^*−/y*^, one-way ANOVA followed by Tukey’s post hoc test, Fig. 5C, E). Overall, these results suggest that Tau reduction promotes MAPK signaling.

A previous study showed that Tau interacted with PTEN and Tau reduction prevented over-activation of the mTOR/PI3K/Akt signaling [8]. Although some previous studies found that the mTOR/PI3K/Akt signaling was over-activated in *Fmr1* KO mice [30, 36, 37], inconsistent results were also reported [32, 38]. Herein, we observed no significant phosphorylation changes of S6, Akt, and mTOR, all of which are indicative of the mTOR/PI3K/Akt signaling activity, in *Fmr1* KO mice when compared to WT mice (Additional file 1: Fig. S1A–D). Nor did we notice that Tau reduction affected the mTOR/PI3K/Akt signaling.

### Tau-targeting ASO rescues autism-like phenotypes in Fmr1 KO mice

**Fig. 6:**
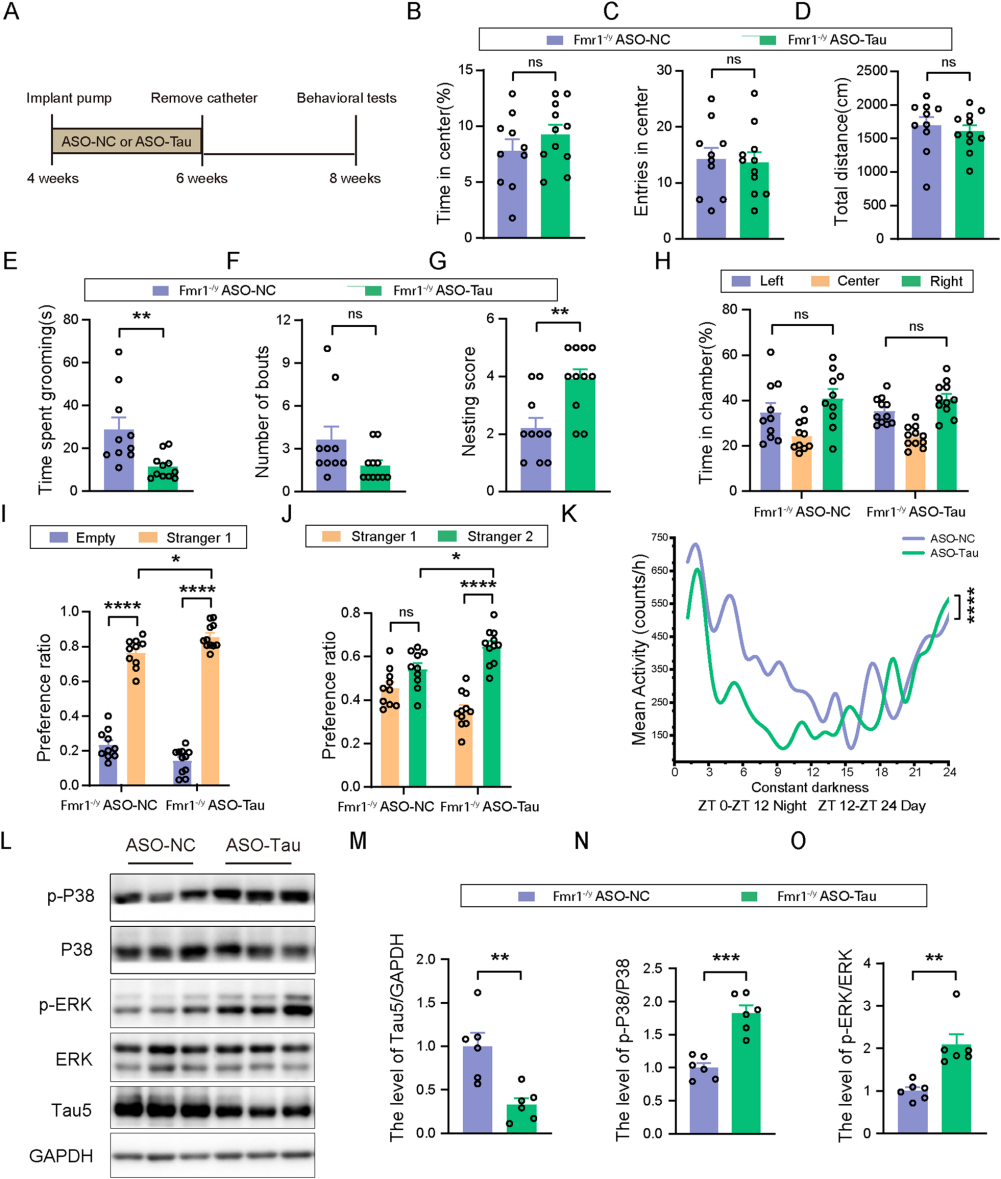
Tau-targeting ASO rescues autism-like phenotypes in *Fmr1*^*−/y*^ mice. **A** ASO treatment paradigm. *Fmr1*^*−/y*^ mice (FVB background, 1-month-old) were treated with 25 μg/d ASO-Tau or ASO-NC via intracerebroventricular infusion for 2 weeks and the catheters were then removed. After another 2 weeks, behavioral tests were performed. **B**–**D** In the open field test, time spent in the center (**B**), center entry numbers (**C**), and total travel distance (**D**) were analyzed. **E**, **F** In the self-grooming test, grooming time (**E**) and bout numbers (**F**) were analyzed. **G** In the nest building test, nesting scores of mice were analyzed. **H**–**J** In the three-chamber social interaction test, time spent in each chamber (**H**), time spent interacting with a Stranger 1 mouse and the empty cage (**I**), and time spent interacting with the Stranger 1 mouse and a Stranger 2 mouse (**J**) were analyzed. **K** In the autonomous wheel-running test, mouse activity was recorded for 5 consecutive days in a constant darkness environment and compared. For behavioral tests in B-J, ASO-NC: *n* = 10; ASO-Tau: *n* = 11. For tests in K, ASO-NC: *n* = 7; ASO-Tau: *n* = 7. **L**–**O** Equal amounts of protein lysates from 2.5-month-old mouse cortical tissues were analyzed by western blotting for indicated proteins (**L**). Total Tau levels were normalized to those of GAPDH for comparison (**M**); and levels of phosphorylated P38 (**N**) and ERK (**O**) were normalized to respective total protein levels for comparison. ASO-NC: *n* = 6; ASO-Tau: *n* = 6. Unpaired t test for (**B**–**G**, **M**–**O**). Two-way ANOVA followed by Bonferroni’s post hoc test for H-J. Two-way repeated-measures ANOVA for K. ns: not significant, *p < 0.05, **p < 0.01, ***p < 0.001, ****p < 0.0001

To further determine whether targeting Tau has therapeutic potential for FXS, we used osmotic pumps to release Tau-targeting ASO (ASO-Tau) and control ASO (ASO-NC) into the lateral ventricles of 1-month-old *Fmr1*^*−/y*^ mice with an FVB background for two weeks. After another two weeks, mice were subjected to various behavioral tests (Fig. 6A). We found that downregulation of Tau by ASO-Tau (*t*_(10)_ = 3.896, *p* = 0.0030, unpaired *t* test, Fig. 6L, M) had no effects on time spent in the center, entries into the center, and total travel distance of mice in the open field test (Fig. 6B–D). In the self-grooming test, ASO-Tau treatment significantly reduced self-grooming time (*t*_(19)_ = 3.133, *p* = 0.0055, unpaired *t* test, Fig. 6E), though not bout numbers (Fig. 6F) of *Fmr1*^*−/y*^ mice. In the nest building test, ASO-Tau mice achieved higher scores than ASO-NC mice (*t*_(19)_ = 3.444, *p* = 0.0027, unpaired t test, Fig. 6G). In the three-chamber social interaction test, neither ASO-Tau nor ASO-NC mice showed preference for each chamber during the habituation phase (Fig. 6H). During the social preference testing phase, although both mice interacted more with a Stranger 1 mouse than an empty cage, ASO-Tau mice had more preference ratios to Stranger 1 than ASO-NC mice (*F*_(1,38)_ = 14.55, *p* < 0.0001 for Stranger 1 versus Empty in all groups, *p* = 0.0143 for Stranger 1 in ASO-Tau versus Stranger 1 in ASO-NC, two-way ANOVA followed by Bonferroni’s post hoc test, Fig. 6I). During the social novelty testing phase, ASO-NC mice showed no preference for a novel Stranger 2 mouse when compared to the familiar Stranger 1. While ASO-Tau mice not only showed preference for Stranger 2 when compared to Stranger 1, but also had more preference ratios to Stranger 2 than ASO-NC mice (*F*_(1,38)_ = 16.21, *p* < 0.0001 for Stranger 2 versus Stranger 1 in ASO-Tau group, *p* = 0.0103 for Stranger 2 in ASO-Tau versus Stranger 2 in ASO-NC, two-way ANOVA followed by Bonferroni’s post hoc test, Fig. 6J). In the autonomous wheel-running test, ASO-Tau mice showed decreased periodic activity when compared to ASO-NC mice (*F*_(1,240)_ = 20.55, *p* < 0.0001, two-way repeated-measures ANOVA, Fig. 6K). Together, these results indicates that ASO-Tau treatment attenuates autism-like behaviors in *Fmr1* KO mice.

Similar to above findings in *Fmr1* KO mice with an FVB;C57BL/6/SV129 mixed background, phosphorylation levels of P38 were decreased (*t*_(8)_ = 1.146, *p* = 0.0360, unpaired *t* test, Additional file 1: Fig. S2A, F), whereas phosphorylation levels of S6, Akt, and mTOR were unaltered (Additional file 1: Fig. S2A–D) in the cortex of *Fmr1* KO mice with an FVB background when compared to those of WT controls. However, ERK phosphorylation was increased in the cortex of *Fmr1* KO mice (*t*(8) = 2.779, *p* = 0.0240, unpaired *t* test, Additional file 1: Fig. S2A, E) with an FVB background but not those with an FVB;C57BL/6/SV129 mixed background. Moreover, we found that ASO-Tau treatment promoted both P38 phosphorylation (*t*_(10)_ = 6.098, *p* = 0.0001, unpaired *t* test, Fig. 6L, N) and ERK phosphorylation (*t*_(10)_ = 4.227, *p* = 0.0018, unpaired *t* test, Fig. 6L, O) but not the mTOR/PI3K/Akt signaling activity (Additional file 1: Fig. S3A–D) in the cortex of *Fmr1*^*−/y*^ mice with an FVB background.

## Discussion

There is an urgent need to identify therapeutic targets for ASDs that affiliate about 1% of the world’s population. Several studies have found that Tau reduction prevents autism-like phenotypes in *Scn1a*^*RX/*+^ and *Cntnap2*^*−/−*^ mice but not in *Shank3B*^−/−^ mice [7–10]. Due to a high heterogeneous etiology of ASDs, whether Tau reduction exerts protection in other types of ASDs deserves scrutiny. FXS is a leading cause of ASDs and results from epigenetic silencing of the *FMR1* gene that encodes FMRP. A variety of approaches to treat FXS, such as peptides [39], small molecule inhibitors[40], and gene therapy [41, 42] are currently under exploration. A previous study found that Tau protein levels were increased in the hippocampi and cerebral cortex of autistic-like rats induced by prenatal exposure of valproic acid [43]. Herein, we also found that Tau expression was increased in the cortex but not the hippocampi of *Fmr1* KO mice. The reason for specific Tau upregulation in the cortex is unclear. One possibility is that FMRP associates with certain factors specifically expressed in the cortex to regulate Tau expression. More importantly, we showed that both genetically reducing and ASO treatment for Tau reduction effectively alleviated social defects, stereotyped and repetitive behavior, circadian rhythm dysregulation, and spine abnormality in *Fmr1* KO mice, indicating that Tau reduction is also a promising strategy for FXS treatment.

One study found that Tau interacted with PTEN to suppress PTEN activity, whereas Tau reduction prevented over-activation of the PI3K/Akt/mTOR signaling pathway in in *Scn1a*^*RX/*+^ and *Cntnap2*^*−/−*^ mice [8]. Although some previous studies suggested that the PI3K/Akt/mTOR signaling pathway was also over-activated in various brain regions of *Fmr1* KO mice and FXS patients [30, 36, 37], there were some other contradictory results. For example, Sawicka et al. and Saré et al. found that mTOR phosphorylation indicative of activation was not altered in the cortex of *Fmr1* KO mice compared to WT controls [32, 38]. Herein, we also found that the PI3K/Akt/mTOR signaling was not over-activated in our *Fmr1* KO mice. Nor did Tau reduction affected the PI3K/Akt/mTOR signaling in *Fmr1* KO mice.

To explore the molecular mechanism underlying the protection by Tau reduction, we carried out RNA sequencing. However, we only found that the expressions of *Per1* and *Gm52433* were altered in *Fmr1*^*−/y*^ versus WT and reversed in *Fmr1*^*−/y*^;*Mapt*^±^ versus *Fmr1*^*−/y*^. *Per1* is an important circadian rhythm gene and we indeed found that Tau reduction not only partially reversed *Per1* expression reduction, but also reversed increased periodic activity in *Fmr1* KO mice. However, since *Mapt*^±^ mice also had decreased *Per1* mRNA expression and abnormal periodic activity when compared to WT mice, the reversal of *Per1* expression is probably only a phenomenon accompanied with the overall improvement by Tau reduction in *Fmr1* KO mice, rather than a responsible molecular mechanism.

Another possibility for Tau reduction to exert protection is that Tau deficiency-affected gene processes/pathways can balance those affected in *Fmr1* KO mice. Among the top 20 enriched GO processes in *Fmr1*^*−/y*^ versus WT and in *Mapt*^±^ versus WT, we found two overlapped GO processes: “response to light stimulus” and “inactivation of MAPK activity.” “Response to light stimulus” is related to circadian rhythm and we already showed that abnormal periodic activity in *Fmr1* KO mice was rescued by Tau reduction. Several previous studies have suggested that FXS is associated with a dysregulation of the MAPK signaling [33, 44, 45]. Indeed, we found that phosphorylation of P38 was significantly decreased in *Fmr1* KO mice with different backgrounds; and this decrease was reversed by Tau reduction. The ERK signaling is another important MAPK pathway and suggested to be involved in FXS [37]. However, different studies generated contradictory results. Although many studies found increased ERK phosphorylation indicative of over-activation [30–32], some other work suggested no change or even a decrease of ERK phosphorylation in *Fmr1* KO mice [33–35]. Herein, we found that although ERK phosphorylation was either unaltered in *Fmr1*^*−/y*^ mice with an FVB;C57BL/6/SV129 mixed background or increased in *Fmr1*^*−/y*^ mice with an FVB background when compared to respective WT controls, it was significantly increased when Tau was reduced in both *Fmr1*^*−/y*^ mice. These results implicate that Tau reduction prevents autism-like phenotypes in *Fmr1* KO mice through modulating the P38 signaling but not the ERK signaling; and this requires further confirmation.

## Limitations

We only investigated male animals in this study. Although FXS afflicts males much more than females [1–3], whether Tau reduction also attenuates ASD-like phenotypes in females deserves further scrutiny. In addition, our study only indicates that Tau ASO treatment attenuates deficits in *Fmr1* KO mice but we do not know whether this is a partial or complete rescue. Further studies using WT mice as controls may help determine this. Moreover, although we found that Tau reduction rescued impaired P38/MAPK signaling in *Fmr1* KO mice, whether this is the responsible molecular mechanism has yet to be further determined.

## Conclusion

In summary, our results highlight the participation of Tau in FXS. By demonstrating that Tau reduction prevents autism-like phenotypes possibly through modulating the P38/MAPK signaling in *Fmr1* KO mice, this study provides strong evidence that Tau is a new target for FXS therapeutics.

### Supplementary Information

**Additional file 1:** Supplementary figures.** Fig. S1**. Tau reduction prevents autism-like behaviors in* Fmr1*^*−/y*^ mice independent of the PI3K/Akt/mTOR pathway.** Fig. S2.** P38 and ERK signaling are altered in the cortex of* Fmr1* KO mice with FVB background.** Fig. S3.** Tau-targeting ASO treatment has no effect on the PI3K/Akt/mTOR pathway.

## Data Availability

The datasets used and/or analyzed in the current study are available from the corresponding author upon reasonable request.

## Abbreviations

ASD: Autism spectrum disorder
ASO: Antisense oligonucleotide
DEGs: Differentially expressed genes
FMRP: *FMR1*-encoded fragile X messenger ribonucleoprotein 1
FXS: Fragile X syndrome
GO: Gene ontology;
ICV: Intracerebroventricular
KO: Knockout
