# Supplementary material for: Tau reduction attenuates autism-like features in Fmr1 knockout mice
Source: Mol Autism. 2023 Nov 7;14:42. doi: 10.1186/s13229-023-00574-1 (PMC10629153; doi:10.1186/s13229-023-00574-1)
Supplement: Supplementary file 1 — Additional file 1: Supplementary figures. Fig. S1. Tau reduction prevents autism-like behaviors in Fmr1−/y mice independent of the PI3K/Akt/mTOR pathway. Fig. S2. P38 and ERK signaling are altered in the cortex of Fmr1 KO mice with FVB background. Fig. S3. Tau-targeting ASO treatment has no effect on the PI3K/Akt/mTOR pathway. [file 13229_2023_574_MOESM1_ESM.docx]

**Supporting information**

**Tau Reduction Attenuates Autism-like Features in *Fmr1* Knockout Mice**


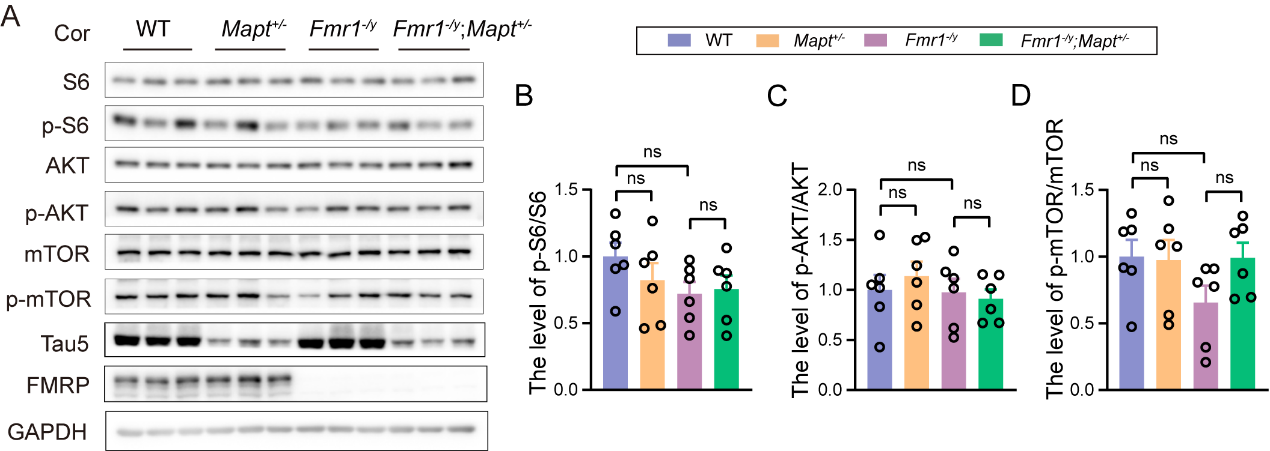


**Fig. S1** Tau reduction prevents autism-like behaviors in *Fmr1^-/y^* mice independent of the PI3K/Akt/mTOR pathway. (A) Equal amounts of protein lysates from mouse (FVB/C57BL/6/SV129 mixed background, 2.5 month-old) cortical tissues were analyzed by western blotting for indicated proteins. (B-D) Levels of phosphorylated S6 (B), AKT (C), and mTOR (D) were normalized to respective total protein levels for comparison. One-way ANOVA with Tukey’s post hoc test. WT: n=6; *Mapt^+/-^*: n=6; *Fmr1^-/y^*: n=6; *Fmr1^-/y^*;*Mapt^+/-^*: n=6. ns: not significant.


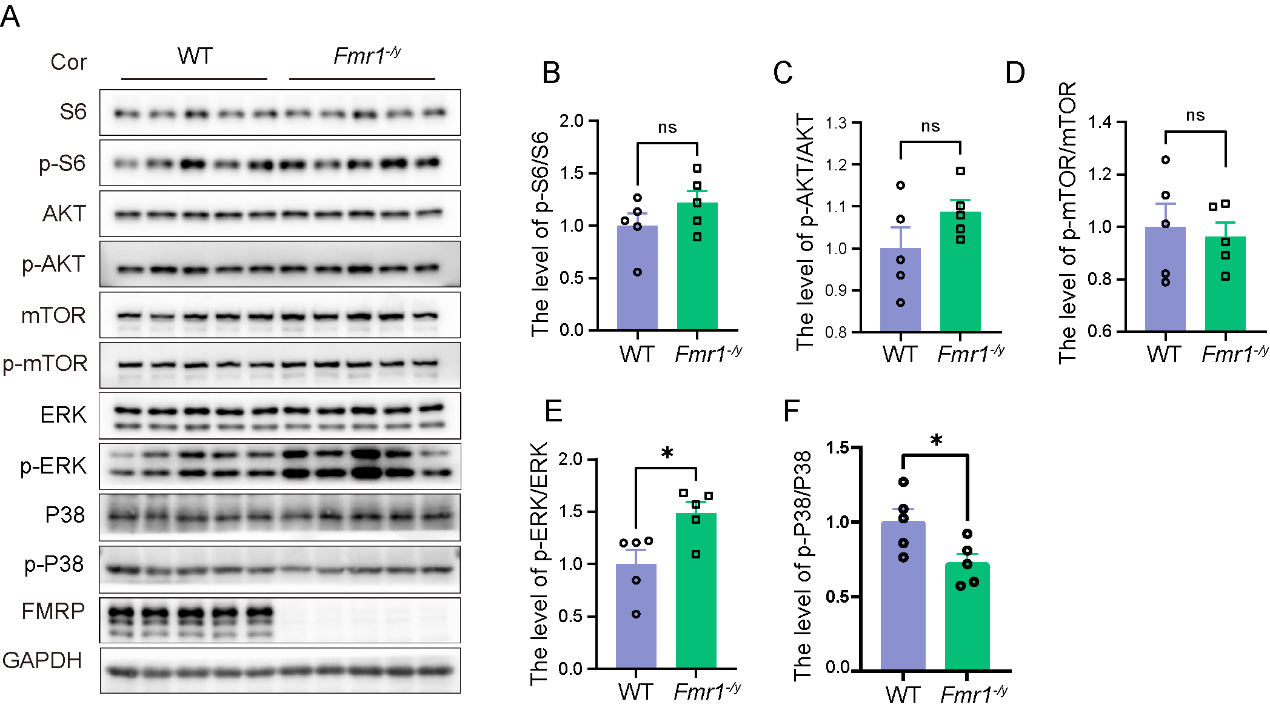


**Fig. S2** P38 and ERK signaling are altered in the cortex of *Fmr1* KO mice with FVB background. (A) Equal amounts of protein lysates from cortical tissues of WT and *Fmr1* KO mice (FVB background, 1.5 month-old) were analyzed by western blotting for indicated proteins. (B-F) Levels of phosphorylated S6 (B), AKT (C), mTOR (D), ERK (E), and p38 (F) were normalized to respective total protein levels for comparison. WT: n=5; *Fmr1* KO: n=5. Unpaired t test. ns: not significant; **p*<0.05.


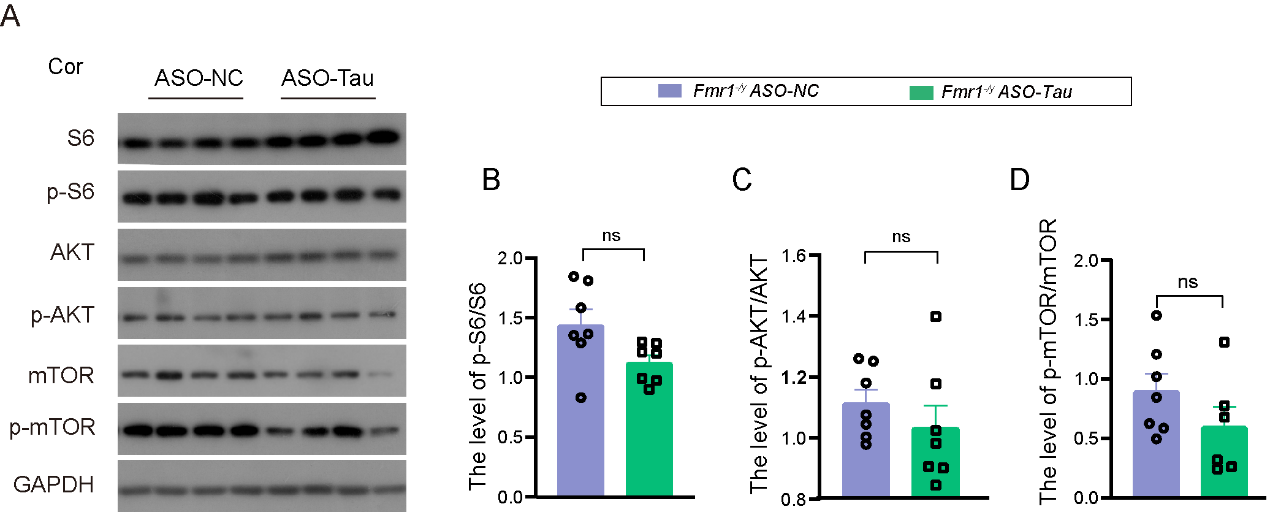


**Fig. S3** Tau-targeting ASO treatment has no effect on the PI3K/Akt/mTOR pathway. (A) Equal amounts of protein lysates from cortical tissues of *Fmr1* KO mice (FVB background, 2.5 month-old) treated with ASO-Tau and ASO-NC were analyzed by western blotting for indicated proteins. (B-D) Levels of phosphorylated S6 (B), AKT (C), and mTOR (D) were normalized to respective total protein levels for comparison. ASO-NC: n=7; ASO-Tau: n=7. Unpaired t test. ns: not significant.
